# Caffeine Acts via A1 Adenosine Receptors to Disrupt Embryonic Cardiac Function

**DOI:** 10.1371/journal.pone.0028296

**Published:** 2011-12-02

**Authors:** Daniela L. Buscariollo, Gregory A. Breuer, Christopher C. Wendler, Scott A. Rivkees

**Affiliations:** Section of Developmental Endocrinology and Biology, Yale Child Health Research Center, Department of Pediatrics, Yale University School of Medicine, New Haven, Connecticut, United States of America; City of Hope National Medical Center and Beckman Research Institute, United States of America

## Abstract

**Background:**

Evidence suggests that adenosine acts via cardiac A1 adenosine receptors (A1ARs) to protect embryos against hypoxia. During embryogenesis, A1ARs are the dominant regulator of heart rate, and A1AR activation reduces heart rate. Adenosine action is inhibited by caffeine, which is widely consumed during pregnancy. In this study, we tested the hypothesis that caffeine influences developing embryos by altering cardiac function.

**Methodology/Principal Findings:**

Effects of caffeine and adenosine receptor-selective antagonists on heart rate were studied *in vitro* using whole murine embryos at E9.5 and isolated hearts at E12.5. Embryos were examined in room air (21% O_2_) or hypoxic (2% O_2_) conditions. Hypoxia decreased heart rates of E9.5 embryos by 15.8% and in E12.5 isolated hearts by 27.1%. In room air, caffeine (200 µM) had no effect on E9.5 heart rates; however, caffeine increased heart rates at E12.5 by 37.7%. Caffeine abolished hypoxia-mediated bradycardia at E9.5 and blunted hypoxia-mediated bradycardia at E12.5. Real-time PCR analysis of RNA from isolated E9.5 and E12.5 hearts showed that *A1AR* and *A2aAR* genes were expressed at both ages. Treatment with adenosine receptor-selective antagonists revealed that SCH-58261 (A2aAR-specific antagonist) had no affects on heart function, whereas DPCPX (A1AR-specific antagonist) had effects similar to caffeine treatment at E9.5 and E12.5. At E12.5, embryonic hearts lacking A1AR expression (A1AR−/−) had elevated heart rates compared to A1AR+/− littermates, A1AR−/− heart rates failed to decrease to levels comparable to those of controls. Caffeine did not significantly affect heart rates of A1AR−/− embryos.

**Conclusions/Significance:**

These data show that caffeine alters embryonic cardiac function and disrupts the normal cardiac response to hypoxia through blockade of A1AR action. Our results raise concern for caffeine exposure during embryogenesis, particularly in pregnancies with increased risk of embryonic hypoxia.

## Introduction

Mature and developing mammals modulate cardiac output through alteration of heart rate and cardiac contractility [1]–[6]. In developing embryos, heart rate changes play an important role in regulating cardiac output [1]–[3]. Factors that alter heart rate thus influence cardiac output and tissue perfusion in early development.

In the embryo and fetus, hypoxia is a stressor with multiple etiologies that include placental insufficiency, maternal smoking, anemia, umbilical cord compression, pre-eclampsia, and living at high altitudes [7]. In mammals, embryonic hypoxia [8], [9] results in intrauterine growth restriction and reduced birth weight [10]. Hypoxia is an important part of the normal development process that drives proper outflow tract formation in the heart, as well as formation of embryonic vessels [11]–[13].

Adenosine is a crucial humoral mediator of functional changes induced by hypoxia [8], [9], [14]. Importantly, hypoxia also plays a key role in inflammation by inducing adenosine production [15]–[17]. Under basal conditions, adenosine levels are low and climb more than 100-fold in hypoxia, ischemia, and inflammation [18]. Adenosine exerts its effects via G protein-coupled receptors that include the A1, A2a, A2b, and A3 subtypes [18]. During embryogenesis, adenosine A1 receptors (A1ARs) are among the earliest expressed G protein-coupled receptors in the heart, with expression observed when the myocardial tube first forms [5]. At the onset of spontaneous cardiac contractions on embryonic day (E) 8.0 in mice, A1AR activation reduces embryonic heart rate [19]. This effect on heart rate becomes more pronounced as gestation progresses [19]. Responsiveness to adrenergic and muscarinic stimuli develops after responsiveness to adenosine, identifying the adenosinergic system as the dominant regulator of early embryonic cardiac function [19].

Recent studies show that adenosine acts via A1ARs in the heart to protect embryos against hypoxia [9], [20]. Embryos lacking cardiac A1ARs that are exposed to hypoxia have decreased viability as compared to embryos expressing cardiac A1ARs [20]. The mechanisms by which cardiac A1AR signaling mediates embryonic protection in hypoxia are not fully known. Our previous work has shown that with the loss of A1AR there is less stabilized hypoxia inducible factor 1 (HIF-1) protein in hypoxia exposed embryos [9]. HIF-1 is a critical hypoxia sensor that is required for normal heart development and mediates a switch from oxidative to glycolytic metabolism to protect cells under hypoxic conditions [13], [21].

Caffeine is a multifunctional compound that influences intracellular calcium levels, inhibits phosphodiesterase activity, and blocks GABA receptors [22]. Yet, at concentrations associated with typical consumption, the predominant effect of caffeine is direct antagonism of adenosine at the receptor level [22]. Caffeine consumption during the first month of pregnancy is reported by 60% of women, with 16% consuming 150 mg or more per day [23]. Although caffeine has not been shown to be teratogenic in humans, consumption during pregnancy is associated with an increased risk of spontaneous abortions and reduced birth weight [18], [24]–[30]. In murine models, exposure to a single dose of caffeine during embryogenesis results in increased body fat and altered cardiac function in adulthood, indicating that caffeine exposure during early development can have long-lasting adverse effects [28].

Considering that adenosine protects the embryo against hypoxia and that A1ARs are the dominant regulator of embryonic heart rate, we postulated that caffeine alters the normal embryonic cardiac response to hypoxia. To test this hypothesis, we examined responses to hypoxia and adenosine receptor blockade on embryonic cardiac activity at two ages spanning the critical period of cardiovascular morphogenesis, E9.5 and E12.5 [31]. We now report that caffeine influences embryonic heart function and impairs normal cardiac responses to hypoxia through blockade of A1ARs.

## Results

### Effects of Caffeine on Embryonic Heart Rate in Room Air

To assess the effects of caffeine on embryonic heart function, whole embryos at E9.5 or isolated hearts at E12.5 were studied. At E9.5, the heart is at an early stage of cardiogenesis, corresponding to only one and a half days after the start of cardiac contractility [19], [31]. E12.5 represents a later stage of cardiogenesis, and this stage is known to be sensitive to adenosine signaling [19], [31].

Before treatments, baseline heart rates were measured following a 1–2 h period. Mean baseline heart rates in the C57Bl/6 embryos were 109.2 beats per minute (BPM) at E9.5 (CI: 106.8–111.6, n = 167) and 124.2 BPM at E12.5 (CI: 120.7–127.6, n = 336). There was a significant age-dependent increase in mean baseline heart rates (p = 0.0001). These values are similar to those observed for murine embryos studied in culture [19], [32], *in vivo* using Doppler ultrasonography, or *in situ* via hysterectomy [33]–[36]. Baseline heart rates between C57Bl/6 comparison groups were not significantly different. In control experiments, heart rates were stable during the time required to conduct these studies.

**Figure 1:**
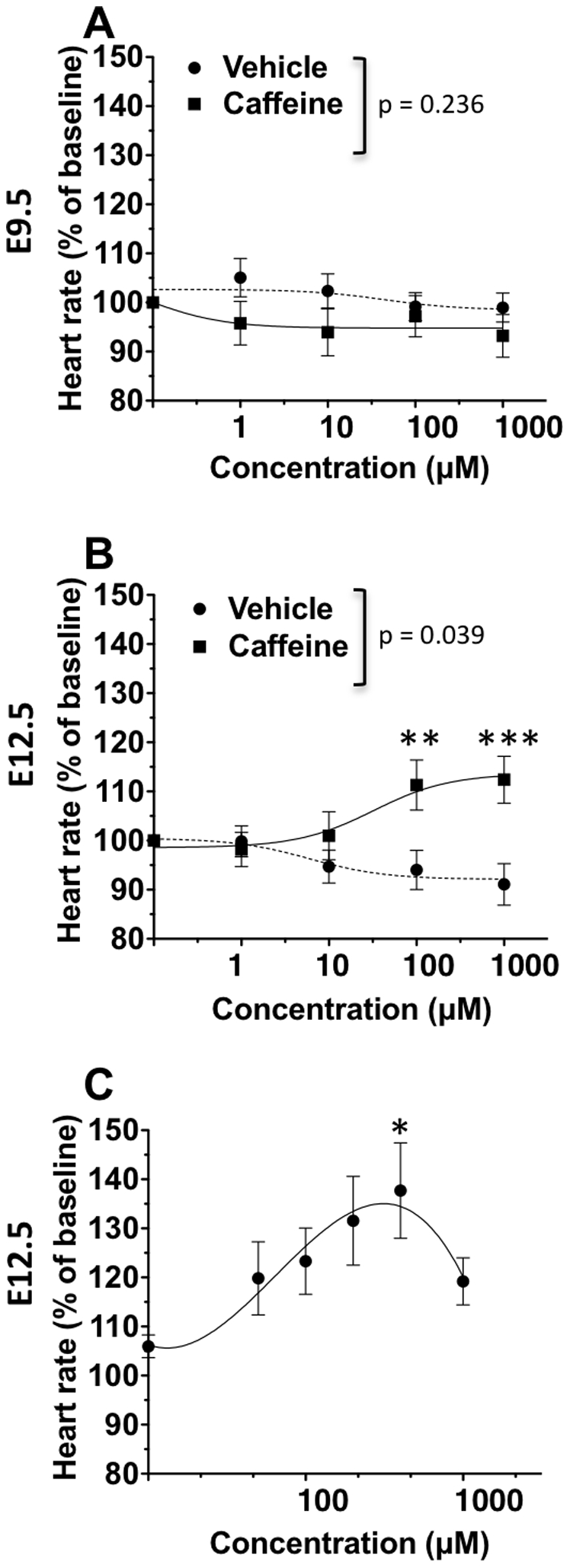
Caffeine concentration-response curves at E9.5 and E12.5. A and B: Specimens were treated with increasing concentrations of caffeine (E9.5, n = 14; E12.5, n = 20) or vehicle (E9.5, n = 13; E12.5, n = 17). In room air, caffeine had no effect at E9.5 (A) but caused a concentration-dependent increase in heart rates at E12.5 (B). C: A concentration-response curve at E12.5 was produced by treating individual hearts with one concentration of caffeine or vehicle (n = 9–10 per concentration). At E12.5, the E_max_ was at 40 µM where caffeine increased heart rates to 137.7% of baseline and the EC_50_ was 118.4 µM. Heart rates were normalized to baseline. Mean ± SEM are shown. X-axes are logarithmically scaled. * p<0.05, ** p<0.01, *** p<0.001 caffeine compared to vehicle.

At E9.5, caffeine treatment in room air had no effect on heart rates, with 1 mM being the highest concentration tested (Fig. 1A). In comparison, caffeine caused concentration-dependent increases in heart rate at E12.5 (Fig. 1B and 1C). Initially, we treated the same hearts with increasing doses of caffeine over time, which indicated that heart rates were affected at doses between 100 nM and 1 mM. Next, we refined this protocol and treated hearts with a single dose of caffeine between 50 nM and 1 mM, and used these results to determine the E_max_ and EC_50_. In the E12.5 hearts, caffeine treatment increased heart rates to an E_max_ of 38% above baseline levels (400 µM), with an EC_50_ of 118.4 µM (Fig. 1C). Interestingly, the magnitude of heart rate elevation decreased at 1 mM relative to the E_max_ (Fig. 1C).

### Effects of Caffeine on the Response to Hypoxia

A1AR action in the heart mediates protective embryonic effects against hypoxia [20], and A1AR agonists decrease heart rate as early as E8.0 [19]. Because caffeine antagonizes adenosine receptors [22], we examined whether caffeine disrupts embryonic cardiac responses to hypoxia by exposing cultured tissues to 2% O_2_ in the presence of 200 µM caffeine or vehicle. This concentration of caffeine was selected based on concentration-response data showing that the compound alters heart function at this concentration.

**Figure 2:**
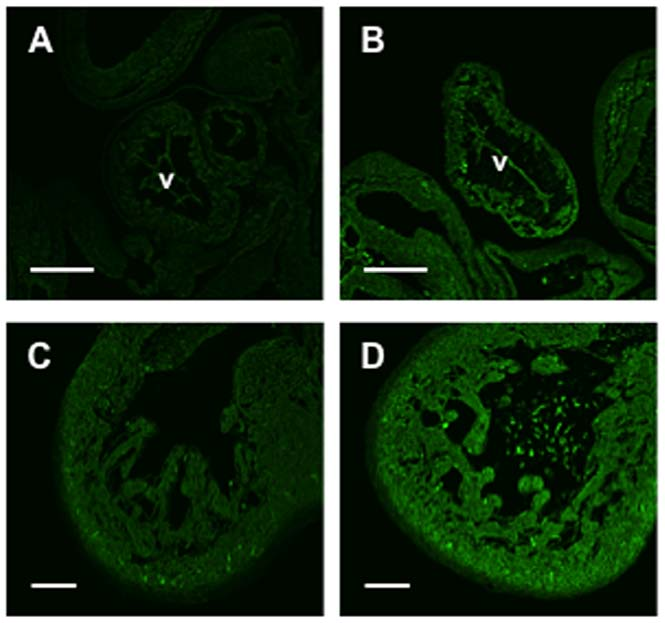
Exposure to 2% O_2_ induces embryonic tissue hypoxia. E9.5 embryos (A and B) and E12.5 hearts (C and D) were incubated with Hypoxyprobe-1 for 1 h under hypoxic (2% O_2_; B and D) or normoxic conditions (A and C). Immunohistochemistry against Hypoxyprobe-1 conjugates (green), which form at <10 mm Hg, demonstrated an increase in tissue hypoxia when embryonic tissues were exposed to 2% O_2_ for 1 h. V, ventricle. Scale bar: 200 µM (A and B), 100 µM (C and D).

To confirm that 2% O_2_ treatment resulted in tissue hypoxia, embryos at E9.5 and hearts at E12.5 were cultured in 2% O_2_ or room air for 1 h in the presence of Hypoxyprobe-1, which forms conjugates when tissue O_2_ levels fall below 10 mm Hg [11]. Analysis of tissue sections showed that 2% O_2_ induced Hypoxyprobe-1 labeling (Fig. 2B and 2D) as compared to normoxic controls (Fig. 2A and 2C).

**Figure 3:**
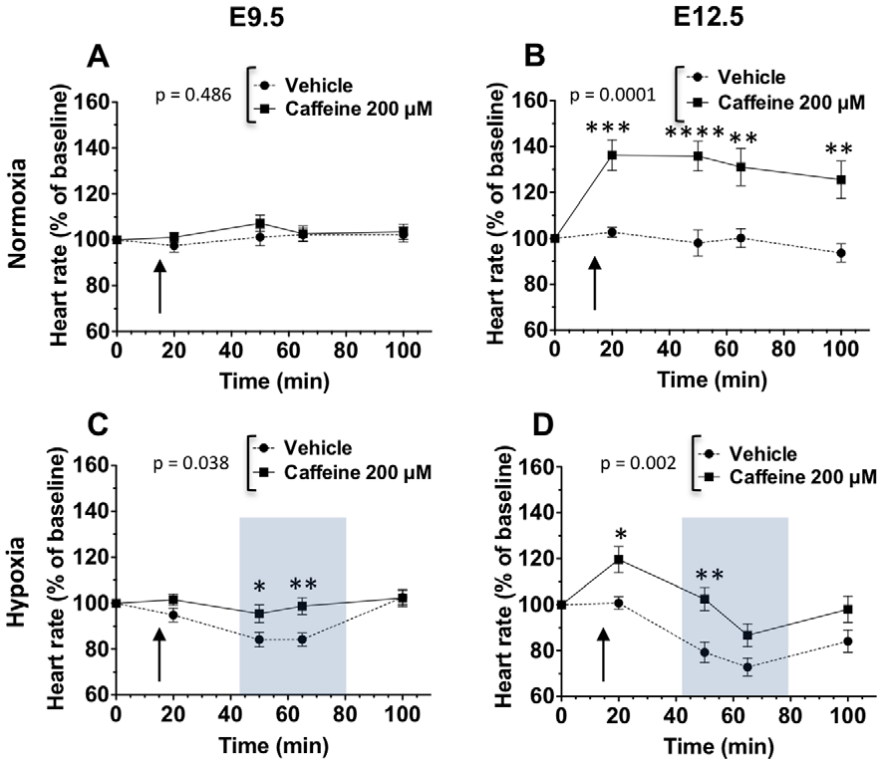
Caffeine effects on embryonic heart function at E9.5 and E12.5 under normoxic and hypoxic (2% O_2_) conditions. In room air, E9.5 embryos (A) and E12.5 hearts (B) were treated with 200 µM caffeine (E9.5, n = 20; E12.5, n = 17) or vehicle (E9.5, n = 24; E12.5, n = 17) at 15 min, as indicated by arrows. At both ages, heart rates were stable for the duration of the experiment, as demonstrated by lack of heart rate variability in vehicle-treated specimens (E9.5, p = 0.394; E12.5, p = 0.263). At E9.5 (A), there were no differences in heart rates between caffeine and vehicle-treated groups. At E12.5 (B), caffeine elevated heart rates above vehicle levels. Following addition of caffeine, heart rates in E12.5 specimens did not vary over time, suggesting the caffeine effects did not significantly diminish in the time required for experiments (p = 0.093). C and D: The effects of hypoxia were determined at both ages in the presence of 200 µM caffeine (E9.5, n = 20; E12.5, n = 22) or vehicle (E9.5, n = 19; E12.5, n = 22). At both ages, heart rates decreased in hypoxia (gray boxes) and increased following recovery in room air (C and D, vehicle, p<0.0001). At E9.5 (C), caffeine treatment completely inhibited hypoxia-mediated decrease in heart rates (p = 0.038). At E12.5 (D), caffeine treatment elevated heart rates and blunted hypoxia-mediated decrease in heart rate (p = 0.002). Heart rates were normalized to baseline. Mean ± SEM are shown. * p<0.05, ** p<0.001, caffeine compared to vehicle at each time point.

Experiments were first conducted in room air to determine whether heart rates were stable over the time required to conduct these experiments (Fig. 3A and 3B). At both ages, heart rates in vehicle-treated specimens did not vary over time (Fig. 3A and 3B). At E9.5, 200 µM caffeine treatment in room air had no effects on heart rates as compared to vehicle (Fig. 3A). At E12.5, hearts treated with 200 µM caffeine in room air exhibited sustained elevations in heart rate above vehicle levels over time (Fig. 3B).

At E9.5 and E12.5, heart rates decreased in hypoxia then increased following recovery in room air for vehicle-treated specimens (Fig. 3C and 3D). This observation is consistent with results from previous studies of E10.5–16.5 murine embryos studied *in situ* via hysterectomy [37]. The magnitude of embryo bradycardia that we observed was greater at E12.5, where heart rates decreased by 27%, as compared to E9.5, where heart rates decreased by 16% (Fig. 3C and 3D). At E9.5, the mean vehicle heart rates returned to baseline after room air recovery, whereas E12.5 heart rates only partially recovered after returning to room air (Fig. 3C and 3D).

At E9.5, caffeine treatment abolished the hypoxia-mediated heart rate reduction (Fig. 3C). At E12.5, caffeine treatment elevated heart rates, resulting in blunted hypoxia-mediated heart rate reductions (Fig. 3D). In the presence of caffeine, E12.5 hearts exposed to hypoxia exhibited delayed heart rate reduction below baseline with a diminished maximal decrease of 13% compared to 27% for the vehicle-treated hearts.

### Adenosine Receptor Gene Expression

**Figure 4:**
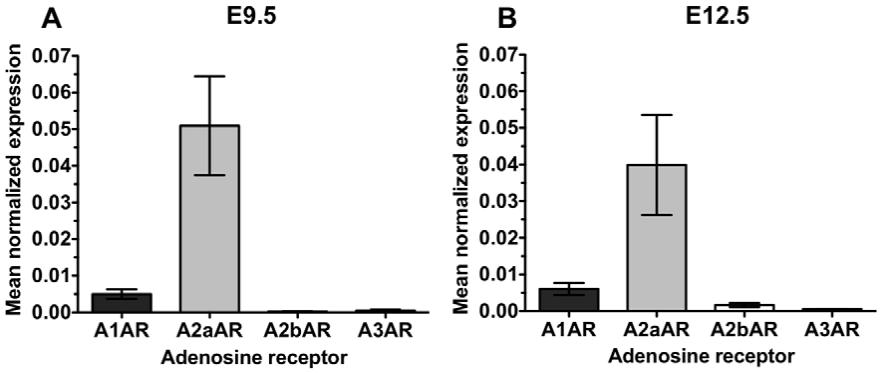
Expression of adenosine receptor subtypes in E9.5 and E12.5 hearts. Real-time PCR analysis was performed on RNA samples extracted from isolated hearts at (A) E9.5 and (B) E12.5. Adenosine receptor gene expression was compared to Rp113a expression to determine the mean normalized expression. Each analysis was performed in triplicate.

We next assessed adenosine receptor gene expression in embryonic hearts. Quantitative real-time PCR analysis was performed using RNA isolated from embryonic hearts at E9.5 and at E12.5. Real-time PCR revealed the presence of *A1AR* and *A2aAR* gene expression in E9.5 and E12.5 embryonic hearts (Fig. 4). In contrast, *A2bAR* and *A3AR* gene expression levels were extremely low at both ages (Fig. 4).

### Effects of Selective Adenosine Receptor Antagonists in Response to Hypoxia

**Figure 5:**
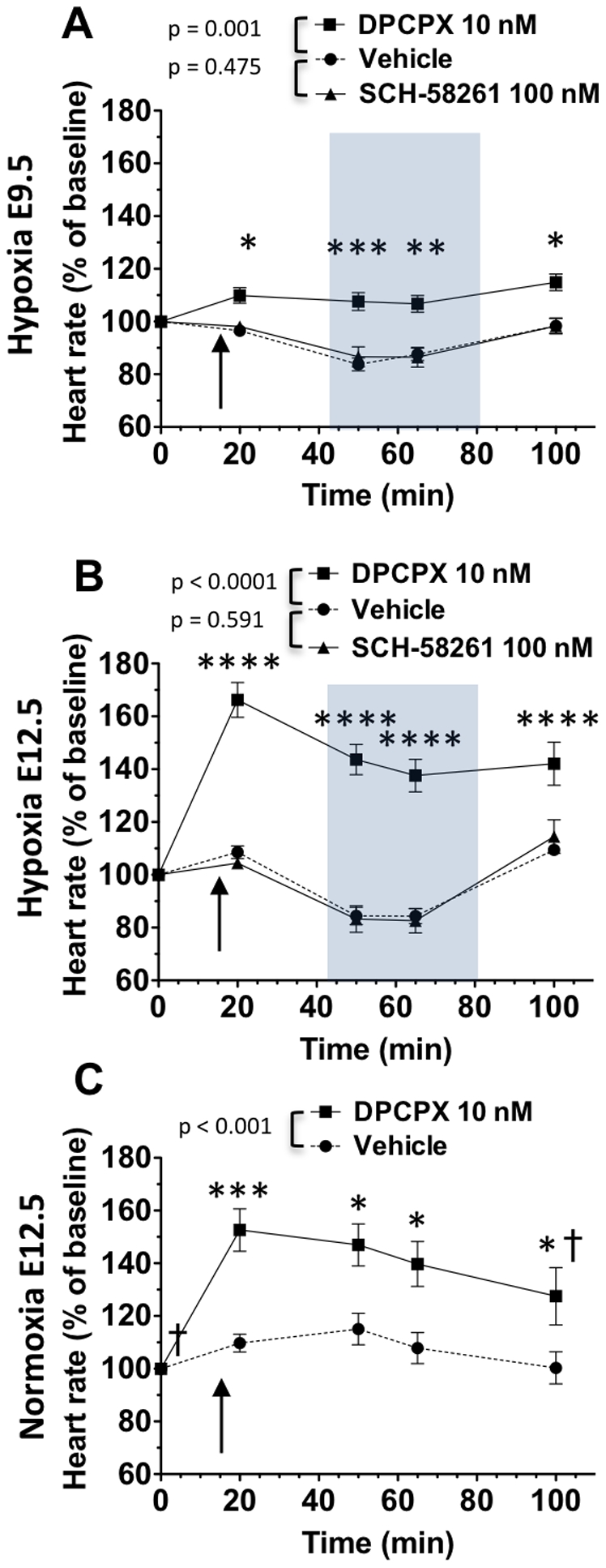
Effects of adenosine receptor-specific antagonists on responses to hypoxia at E9.5 and E12.5. A and B: Specimens were treated with DPCPX, an A1AR-specific antagonist at 10 nM (A: E9.5, n = 15; B: E12.5, n = 23), SCH-58261, an A2aAR-specific antagonist at 100 nM (A: E9.5, n = 15; B: E12.5, n = 19), or vehicle (A: E9.5, n = 27; B: E12.5, n = 42) at 15 min (arrows), and exposed to hypoxia (gray boxes). At both ages, heart rates were not affected by SCH-58261 treatment (A and B). In contrast, at both ages DPCPX elevated heart rates above vehicle levels in normoxic conditions and inhibited hypoxia-mediated heart rate decrease below baseline (A and B). Although E12.5 heart rates did not fall below baseline in hypoxia, heart rates decreased compared to levels following DPCPX addition in room air (p<0.01 and p<0.0001, for t = 50 min and t = 65 min compared to t = 20 min, respectively, B). C: Because heart rates in E12.5 DPCPX-treated hearts significantly decreased in hypoxia, responses to DPCPX were examined over time in normoxia. E12.5 hearts were treated with 10 nM DPCPX (n = 19) or vehicle (n = 19). DPCPX- mediated heart rate elevation did not significantly diminish over time compared to levels at t = 20 min. Heart rates were normalized to baseline. Mean ± SEM are shown. * p<0.05, ** p<0.001, DPCPX compared to vehicle at each time point; † p<0.05 compared to DPCPX at t = 20 min.

Because caffeine is a non-selective adenosine receptor antagonist, we examined the effects of A1AR and A2aAR specific antagonists on heart rates. At both E9.5 and E12.5, embryonic heart rates in room air did not change following the addition of SCH-58261, an A2aAR specific antagonist (Fig. 5A and 5B), with the highest concentration tested being 10 µM (E12.5 vehicle, n = 17; E12.5 SCH-58261, n = 15). Heart rate reductions in hypoxia were similar between SCH-58261 and vehicle-treated groups (Fig. 5A and 5B).

In contrast to that seen with an A2AR antagonist, DPCPX treatment affected embryonic heart function at both ages. In room air (t = 20 min), DPCPX caused significant elevations in heart rates that were more pronounced at E12.5 (67% increase) than at E9.5 (10% increase, Fig. 5A and 5B). At both ages, unlike vehicle-treated groups, heart rates did not decrease below baseline when exposed to DPCPX in hypoxia (Fig. 5A and 5B). Although E12.5 heart rates did not fall below baseline in hypoxia in the presence of DPCPX, they decreased when exposed to hypoxia (Fig. 5B). In room air, the heart rate elevation observed in the DPCPX-treated E12.5 hearts diminished over time. This decrease was not statistically significantly different as compared to t = 20 min levels (Fig. 5C), suggesting that heart rate reductions in hypoxia are attributed to low O_2_ levels and not to waning effects of DPCPX.

### Effects of A1AR Loss on Embryonic Response to Caffeine and Hypoxia

To further characterize the role of A1ARs in the response to caffeine and hypoxia, embryos from global A1AR knockout mice were studied. At E9.5, baseline heart rates did not differ by A1AR genotype (Table 1). In contrast, E12.5 A1AR −/− specimens had significantly higher baseline heart rates compared to A1AR +/+ and A1AR +/− hearts (Table 1). There were no differences in baseline heart rates between E12.5 A1AR +/+ and A1AR +/− hearts (Table 1).

**Table 1:** Mean baseline heart rates in A1AR transgenic embryos.

	Genotype	BHR (in BPM)	CI (95%)	p	n
**E9.5**	A1AR +/+	122.9	115.7–130.2	0.71	25
	A1AR +/−	126.7	121.3–132.1		50
	A1AR −/−	125.9	115.1–136.7		15
**E12.5**	A1AR +/+	146.7	129.5–164.2	0.01	20
	A1AR +/−	150.3	139.5–161		45
	A1AR −/−	171.9 * †	162.2–181.6		31

Statistical analyses performed were repeated-measures one-way ANOVA followed by Bonferroni *post hoc* test.

*p<0.05, compared to A1AR +/+;

† p<0.05, compared to A1AR +/−. BHR, baseline heart rate; BPM, beats per minute; CI, confidence interval.

**Figure 6:**
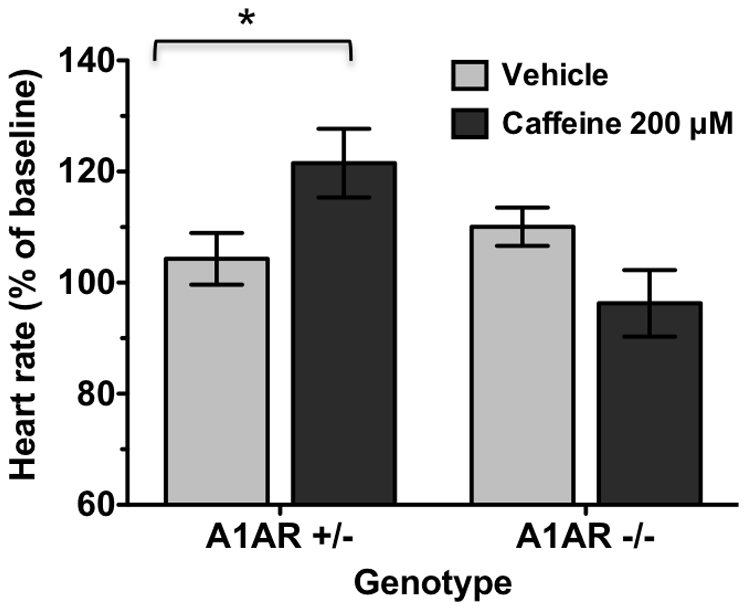
Effects of A1AR expression on response to caffeine at E12.5. Isolated hearts lacking A1ARs (−/−) were treated with 200 µM caffeine (n = 16) or vehicle (n = 10). Hearts from A1AR +/− littermates were treated with 200 µM caffeine (n = 18) or vehicle (n = 20). Caffeine treatment elevated heart rates in cultured hearts expressing A1ARs (p = 0.03). In hearts lacking A1ARs, caffeine treatment had no significant effect on heart rate (p = 0.101). Heart rates were normalized to baseline. Mean ± SEM are shown. * p = 0.03 caffeine compared to vehicle, unpaired Student's t-test.

Pharmacological studies indicated that caffeine response profiles were similar to those of A1AR-specific antagonists. To assess if the positive chronotropic effects of caffeine at E12.5 are mediated through blockade of A1ARs, effects of 200 µM caffeine in hearts lacking A1AR expression were examined. Following caffeine treatment, heart rates in A1AR −/− specimens were not significantly different from vehicle-treated specimens (Fig. 6). Heart rates in hearts expressing A1ARs (+/−) increased significantly compared to vehicle-treated specimens by 22% above baseline (Fig. 6). These results indicate that effects of caffeine on E12.5 heart rate in room air are mediated through A1ARs.

**Figure 7:**
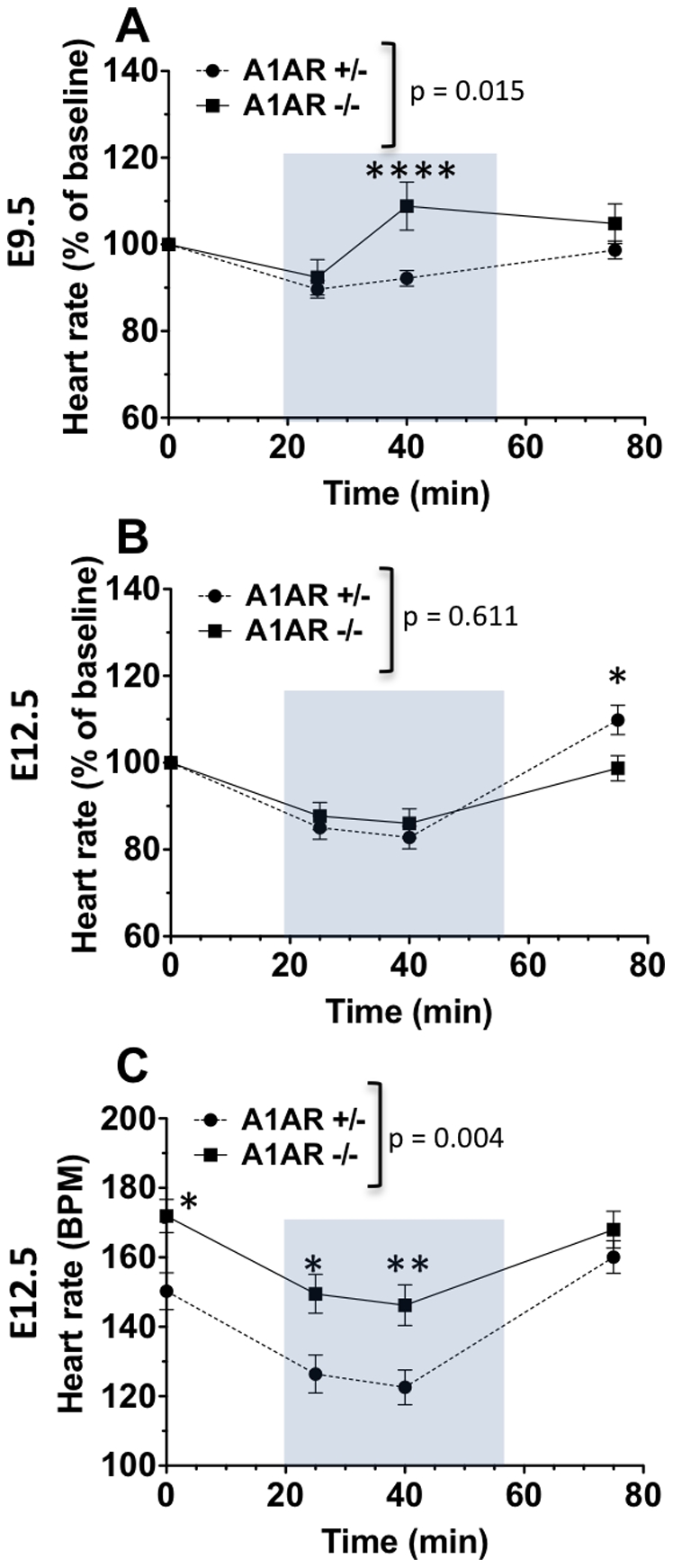
Effects of A1AR expression on response to hypoxia at E9.5 and E12.5. A: At E9.5, responses to hypoxia in A1AR −/− (n = 15) and A1AR +/− (n = 50) embryos were studied. In hypoxia, E9.5 A1AR +/− heart rates decreased then returned to baseline levels following recovery in room air (p<0.0001 and p<0.01, for t = 25 and t = 40 compared to t = 0, respectively). E9.5 A1AR −/− responses to hypoxia were impaired as compared to controls, where responses were characterized by initial heart rate reductions followed by paradoxical heart rate elevations above A1AR +/− levels. B: At E12.5, hypoxia decreased heart rates below baseline in both hearts lacking A1ARs (n = 31) and those of control A1AR +/− littermates (n = 45; p<0.0001). When heart rates were analyzed as a percent of baseline levels there was no statistically significant difference in overall response among the different genotypes (p = 0.612). Because lack of A1ARs resulted in increased baseline heart rates when compared to those of control (p<0.05), non-normalized was also analyzed (C). This observation revealed that although hypoxia caused proportional decreases in heart rate independent of A1ARs, without A1ARs heart rates remained elevated above controls (p = 0.004). Mean ± SEM are shown. * p<0.01, ** p<0.0001, A1AR−/− compared to A1AR+/− at each me point.

To further probe the role of A1ARs on hypoxia-induced bradycardia, effects of hypoxia were examined in A1AR deficient embryos. At E9.5, the response to hypoxia was affected by genotype (Fig. 7A). In hypoxia, heart rates of A1AR +/+ and A1AR +/−hearts had maximal decreases of 12% and 10% (Fig. 7A), respectively. Response profiles of A1AR +/+ and +/− hearts did not differ (p = 0.502, data not shown). In contrast, E9.5 A1AR −/− embryos exhibited impaired hypoxia-mediated heart rate reductions compared to controls. In hypoxia, heart rates of A1AR −/− hearts slightly decreased initially and then increase 9% above baseline levels 20 min after hypoxia exposure (t = 40 min) before returning to baseline following recovery in room air (Fig. 7A). This response profile for the E9.5 A1AR −/− hearts may be caused by the fact that E9.5 hearts are not as sensitive to hypoxia compared to older E12.5 hearts, and therefore it takes longer to observe the effects of A1AR loss on heart rates.

At E12.5, heart rates decreased in hypoxia independent of genotype (Fig. 7B). In hypoxia, heart rates of A1AR +/+ hearts had a maximal decrease of 20% (data not shown), those of A1AR +/− hearts decreased by 17%, and those of A1AR −/− hearts decreased by 13% (Fig. 7B). When heart rates were analyzed as a percent of baseline levels, there were no statistically significant differences in responses to hypoxia among the different genotypes (Fig. 7B), indicating that hypoxia resulted in proportional decreases in heart rate.

**Figure 8:**
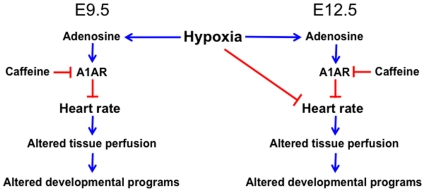
Schematic of influences of adenosine and hypoxia on embryonic heart rate regulation. Hypoxia induces increased levels of adenosine, which in turn activate A1ARs to lower heart rate. Reduction in heart rate will affect embryonic tissue perfusion, which in turn will influence embryonic development. At E9.5, effects of hypoxia on heart rate appear to be exclusively mediated via A1ARs. At E12.5, hypoxia also can influence heart rate independent of A1ARs. Caffeine, an adenosine antagonist, will perturb this physiological cascade.

Because A1AR−/− baseline heart rates are higher than A1AR+/+ and A1AR+/− littermates, we compared heart rates that were not normalized to baseline rates (Fig. 7C). Hypoxia decreased heart rates below baseline in A1AR +/+ and A1AR +/− hearts, but A1AR −/− heart rates never fell below the baseline heart rate of A1AR+/+ hearts in room air (Fig. 7C). These data indicate that at E9.5 hypoxia-induced bradycardia is mediated by A1AR signaling, whereas at E12.5 hypoxia also induces bradycardia through non-A1AR pathways (Fig. 8).

## Discussion

Evidence shows that adenosine acts via A1ARs to protect embryos from hypoxia [9], [20]; however the mechanisms by which A1ARs confer embryo protection remain poorly understood [9], [20]. To address this issue, we investigated whether A1AR signaling modulates embryonic cardiac function, in responses to hypoxia. We now show that hypoxia decreases embryonic heart rate through A1ARs in early embryonic hearts (E9.5) and that this normal physiological response is blocked by caffeine via blockade of adenosine action on A1ARs. In addition, we demonstrate that inhibition of A1AR signaling, either by drug treatment or gene knockout, in older hearts (E12.5) leads to elevated heart rates. In contrast to E9.5, E12.5 hearts have reduced heart rates in response to hypoxia even in the presence of A1AR antagonists. This indicates that older hearts have other non-A1AR pathways that can mediate hypoxia induced bradycardia (Fig. 8).

Previous studies examining murine embryos *in situ* via hysterectomy demonstrated that hypoxia reduces heart rates and that this effect is greater in older embryos [37], [38]. Our studies similarly showed that E12.5 embryonic hearts had a greater reduction in heart rate in response to hypoxia as compared to E9.5 hearts. These results show that intrinsic embryonic and/or cardiac factors contribute to hypoxia-mediated bradycardia during embryogenesis rather than maternal or placental effects.

The developing cardiovascular system is vulnerable to hemodynamic stress [6], [39]–[42] and abnormal nutritional support [43]–[45]. Therefore, altered embryonic heart rate will have direct consequences on the developing embryo and fetus [46]–[48].

In adult and embryonic mice, A1AR activation has been shown to potently reduce heart rates [19], [49]. We observed that the A1AR-specific antagonist DPCPX completely abolished hypoxia-mediated heart rate reductions at E9.5 and prevented heart rate reductions in E12.5 hearts from going below baseline heart rates. In comparison, no effects of A2aAR antagonism were seen. These observations highlight the dominant role for A1ARs in mediating adenosine action in embryos. Further supporting this notion, at E9.5, embryos lacking A1ARs had impaired responses to hypoxia characterized by an increase in heart rate after 20 min compared to a decrease in heart rate observed in A1AR+/− littermates. At E12.5, A1AR −/− heart rates failed to decrease to levels comparable to those of littermates expressing A1ARs when exposed to hypoxia. Although A1AR−/− hearts reduced their heart rate in response to hypoxia, there may still be a negative effect on heart development if the decrease is not enough to trigger a protective response to hypoxia.

Significant heart rate elevations were observed at caffeine concentrations as low as 100 µM, which is comparable to caffeine levels produced with common human caffeine ingestion [22]. At higher doses of caffeine of 1 mM, we saw diminished chronotropic effects. It is possible that the effects of caffeine observed at 1 mM reflect non-adenosine antagonism mechanisms. Positive chronotropic effects of caffeine have been observed in human embryonic and fetal hearts in culture [50]–[53]. Yet, studies examining the *in vivo* hemodynamic effects of caffeine in human pregnancy have been inconsistent, and have shown that caffeine decreases or has no effect on fetal heart rate [54]–[57]. In addition, it has been demonstrated that adult mice heterozygous for both A1 and A2a adenosine receptors mimic mice treated long-term with caffeine, effects include decreased heart rate [58]. In mice, daily caffeine doses of 10 mg/kg were associated with embryonic size reduction that were thought to be mediated through A2aARs, but no changes in heart rates were observed [59]. That study, though, was performed under isofluorane sedation [59], and may have masked effects of caffeine on heart rates. Considering the challenges associated with *in vivo* models, our embryonic tissue cultures allowed direct examination of effects of caffeine and hypoxia on embryonic heart rate, revealing direct effects on embryos and hearts.

It is important to note that although our focus is on A1ARs and a prominent A1AR effect was observed, other adenosine receptor subtypes may play a role in mediating effects of hypoxia. A2bARs and other signaling pathways that are implicated in hypoxia responses have been observed, with hypoxia inducing A2bAR expression along with enzymes that increase adenosine production [60].

In humans, effects of caffeine on the embryonic [54], [55], [57], uteroplacental [55], [56], and maternal [54]–[57], [61], [62] circulations have been observed. These results have indicated that caffeine increases uteroplacental and fetal vascular resistance, possibly through both direct antagonism of vascular adenosine receptors and increased release of catecholamines [55]–[57]. With the exception of one study [57], most of these reports examined effects of caffeine on second or third trimester pregnancies. In comparison, our study was performed at stages that are equivalent to the first trimester in humans.

We believe that our studies are clinically relevant for several reasons. First, we observed that baseline heart rates were very similar to those obtained *in vivo* in murine embryos [4], [33]–[35]. Second, although heart rate is dependent on stroke volume and thus preload, afterload, and contractility via neural feedback loops in mature mammals, the sympathetic and parasympathetic neurons first innervate the heart much later in gestation than the ages we studied [63], [64]. Third, we are unaware of other systems in which individual murine embryos can be treated with specific concentrations of drugs for a prolonged period independent of maternal or placental physiologic effects.

In summary, we demonstrated that hypoxia decreases heart rates in E9.5 and E12.5 embryos, and that caffeine disrupts this response by elevating embryonic heart rates through inhibition of A1AR action. These results raise concern for caffeine exposure during embryogenesis, especially in pregnancies with increased risk of embryonic hypoxia, including placental insufficiency, anemia, maternal smoking and living in high altitudes [7]. Our observations are consistent with other reports suggesting that adverse effects of caffeine ingestion are strongest during early pregnancy [30]. Additional studies on the effects of caffeine exposure on developing embryos and embryonic heart function are needed.

## Materials and Methods

### Animals

The Institutional Animal Care and Use Committee (IACUC) of Yale University approved all experiments conducted on animals (protocol #2007-11136). The inbred mouse strain C57Bl/6 (Charles River Laboratories, Wilmington, MA) and A1AR-deficient mice (A1AR −/−) were studied [65]. Timed matings were used to obtain the appropriate age embryos, with E0.5 designating the day a vaginal plug was observed. To genotype A1AR transgenic mice, genomic DNA extraction and PCR were performed as described [66].

### Tissue Cultures

Embryos and hearts were cultured as described [19]. Hysterectomy was performed under sterile conditions, and uteri were rinsed in Dulbecco's PBS (DPBS) with 2 mM MgCl_2_. Embryonic age was determined by morphology and somite number [67]. E9.5 embryos were cultured intact with yolk sacs removed and pericardia punctured. At E12.5, isolated hearts were studied. Embryonic tissues were transferred into individual wells containing DMEM with 10% fetal bovine serum (Fetal Clone II, Hyclone Laboratories, Logan, UT) and 50 mM HEPES, pH 7.3, buffer. Specimens were incubated at 37°C in a 5% CO_2_ –room air incubator for 1–2 h prior to conducting tissue hypoxia studies and heart rate analyses.

### Tissue Hypoxia Assessment

To assess if there was embryonic tissue hypoxia associated with 2% O_2_ exposure, C57Bl/6 embryonic tissues were cultured with 200 µM Hypoxyprobe-1 (pimonidazole; Chemicon International, Temecula, CA). Hypoxyprobe-1 forms protein adducts in O_2_ levels <10 mm Hg that can be detected by immunostaining [11]. Following a 1 h incubation period, embryos or hearts were exposed to 2% O_2_ in a Plexiglass chamber (Biospherix, Redfield, NY) for 1 h or left in the incubator for 1 h for room air controls. O_2_ levels were maintained at 2% with a regulator (Biospherix) by introducing 95% nitrogen with 5% CO_2_ into the chamber. Chamber temperature was maintained between 35°C and 37°C with a heating cord. Tissues were fixed in 4% paraformaldehyde, embedded in paraffin, and sectioned. Conjugates were visualized with a monoclonal antibody to Hypoxyprobe-1 (Chemicon International, Temecula, CA) followed by a goat anti-mouse secondary antibody conjugated to Alexa488 (Santa Cruz Biotechnology, Santa Cruz, CA), as described [9]. Images were captured with an Olympus Fluoview laser scanning confocal microscope.

### Heart Rate Studies

#### Heart rate assessment

Using the chamber described above, heart rate measurements were performed through direct visualization with a dissecting microscope over 15-second intervals by a single individual who was blinded to treatment conditions. Measurements were performed in triplicate per sample at each time point.

#### Caffeine Concentration-Response Studies

To generate concentration-response curves at E9.5 and E12.5 under normoxic conditions, individual specimens of the C57Bl/6 strain were treated with vehicle or increasing concentrations of caffeine (Sigma-Aldrich, St. Louis, MO). Six to 12 specimens were analyzed per experiment. Following a 1–2 h initial incubation period, the baseline heart rate for each specimen was measured. Individual specimens were treated with increasing concentrations of caffeine (1 µM, 10 µM, 100 µM, and 1 mM) or vehicle without changing the media, and heart rates were reassessed after 2 min. In addition, at E12.5, individual hearts were treated with vehicle or a single concentration of caffeine (50 µM, 100 µM, 200 µM, 400 µM, or 1 mM) to refine the concentration-response curves.

#### Effects of Hypoxia and Adenosine Receptor Antagonists on Heart Rate

Heart rates of C57Bl/6 embryos were assessed following hypoxia exposure and/or treatment with adenosine receptor antagonists. Cultures were treated with 200 µM caffeine. This concentration was selected based on our caffeine concentration-response data (see Results). In addition, some cultures were treated with 10 nM 1,3-dipropyl-8-cyclopentylxanthine (DPCPX; Sigma-Aldrich, St. Louis, MO), an A1AR-specific antagonist, and 100 nM SCH-58261 (Sigma-Aldrich, St. Louis, MO), an A2aAR-specific antagonist. The concentrations were chosen based on previous reports [68].

Heart rate experiments began after an initial 1–2 h incubation period following dissections. Plates containing 6–12 specimens were placed in the chamber and baseline heart rates were measured (time [t] = 0 min). Drugs or vehicle were added to each well in a blinded fashion and heart rates were reassessed after 5 min (t = 20 min). The chamber oxygen levels were lowered to 2.0%. This O_2_ level was selected based on our Hypoxyprobe-1 studies that showed increased tissue hypoxia while embryonic cardiac function was preserved. Heart rates were measured after 5 min in 2.0% O_2_ (t = 50 min) and 20 min in 2.0% O_2_ (t = 65 min). Finally, heart rates were reassessed after a 10 min recovery period in room air O_2_ (t = 100 min). In control experiments to show that embryonic heart function in culture is stable over time, heart rates were assessed at these time points without hypoxia or caffeine treatment.

#### Heart rate analysis of A1AR knockout mice

Effects of caffeine and hypoxia on A1AR knockout mice was determined as described above for C57Bl/6 mice.

### Real-Time RT-PCR Analysis for Adenosine Receptors

Gene expression was determined for all four adenosine receptor subtypes in isolated hearts from E9.5 and E12.5 C57Bl/6 embryos, as described [69]. Isolated hearts were collected. Tissue was rinsed in DPBS, placed in 1.5 ml microcentrifuge tubes, flash frozen in liquid nitrogen, and stored at −80°C until RNA isolation. Total RNA was extracted according to the manufacturer's instructions with the RNeasy Plus Kit (Qiagen, Valencia, CA), as described [69]. RNA was isolated from three separate pools of at least 15 hearts each. 1 µg of RNA was reverse transcribed using the iScript cDNA Synthesis Kit (Bio-Rad Laboratories, Hercules, CA), as reported [69]. Each real-time PCR reaction contained IQ SYBR Green Super Mix (Bio-Rad, Hercules, CA), 50 ng cDNA, and 0.5 µM of each primer in a 20 µl reaction volume. PCR was performed at 55°C for annealing in an Opticon 2 DNA Engine PCR machine (Bio-Rad). All primers were designed against mouse sequence (Rp113a housekeeping gene: forward 5′- ATGACAAGAAAAAGCGGATG – 3′, reverse 5′ – CTTTTCTGCCTGTTTCCGTA – 3′; A1AR: forward 5′ – GTCCTGGTATGTGACCAAGC – 3′, reverse 5′ – CTGAGGCTCTGGGTGAACTA 3′; A2aAR: forward 5′ – GCAGAACGTCACCAACTTCT – 3′, reverse 5′ – ATGGCGATGTATCTGTCGAT – 3′; A2bAR: forward 5′ TGCATGCCATCAACTGTATC – 3′, reverse 5′ – TGGAAACTGTAGCGGAAGTC; A3AR: forward 5′ – AGCGAGCTCTCAAAATAGCA 3′, reverse 5′ – ATAAAATGCACGCGTCTCTC 3′) and were obtained from RealTimePrimers.com (Elkins Park, PA). Each real-time PCR experiment was performed in triplicate. PCRs were analyzed by mean normalized expression using Microsoft Excel, with Rp113a as the control gene. DNA contamination was assessed by a “no RT control” real-time PCR reaction, no DNA contamination was observed.

### Statistical Analysis

Heart rates were measured in triplicate and recorded in a spreadsheet (Microsoft Excel) where average heart rate in beats-per-minute (BPM) were calculated. To standardize heart rates from all studies, heart rates were recorded as percent change from baseline using the formula: percent change = heart Rate/baseline×100. Descriptive statistics including 95% confidence intervals (CI) were calculated using Excel (Microsoft, Redmond, WA). Concentration-response curves were generated using GraphPad Prism version 5 (GraphPad Software Inc., San Diego, CA). The EC_50_, defined as the drug concentration that caused heart rates to increase to levels halfway between baseline and maximum levels, was obtained from these curves where the lowest concentration corresponded to the addition of vehicle. The maximal mean increase in heart rate from basal levels was defined as E_max_. Differences in response profiles over time between treatment or genotype groups were analyzed with repeated measures two-way ANOVA, differences within groups at different time points were analyzed with repeated measures one-way ANOVA, and differences in mean baseline heart rates were analyzed with unpaired Student's t-test assuming equal variances or one-way ANOVA. Bonferroni's tests were used for *post hoc* analyses. Data are presented as mean +/− the standard error of the mean (SEM). Two-tailed *p*<0.05 was considered to be statistically significant.
